# Additional Evidence Fails to Associate Variation in *KCNE4* With Equine Anhidrosis

**DOI:** 10.1002/age.70109

**Published:** 2026-04-26

**Authors:** Jessica L. Petersen, Carrie J. Finno

**Affiliations:** ^1^ Department of Animal Science University of Nebraska‐Lincoln Lincoln Nebraska USA; ^2^ Department of Population Health and Reproduction University of California, Davis School of Veterinary Medicine Davis California USA

**Keywords:** genetic testing, genome‐wide association, horse, linkage disequilibrium, replication, sweat

## Abstract

A prior genome‐wide association (GWA; *N* = 200) including Thoroughbreds and stock horses implicated chromosome 6 (NC_009149.3) in owner‐reported equine anhidrosis. A missense variant in *KCNE4* (NC_009149.3:g.11813731A>G) was proposed as a risk allele, although its association with anhidrosis was not reported. Variant annotation and protein modelling in the original study suggested the G allele conferred risk. We reported no association of the G allele with anhidrosis in 50 horses phenotyped by an intradermal terbutaline sweat test (ITST); all horses produced sweat regardless of genotype. It later appeared the A allele was instead suggested to confer disease risk. To reassess this, we genotyped 20 ITST‐tested Thoroughbreds including 9 with partial or complete anhidrosis. The *KCNE4* A allele was not associated with phenotype when analyzed as a binary trait (*p* = 0.16) or when classifying affected horses as having either partial or complete anhidrosis (*p* = 0.21). The locus was also uninformative in four clinical cases (AA = 1, AG = 1, GG = 2). Reasoning that a true risk allele should be in linkage disequilibrium (LD) with the associated GWA SNV (AX‐103822151; rs68656009), we evaluated whole‐genome sequence (*N* = 2) from the initial publication. Both case and control were homozygous AA at the putative risk locus; the case was heterozygous at the GWA SNV. In public data (*N* = 897), LD between loci was low (r^2^ = 0.19). In 369 Thoroughbreds, LD was 0.58. Restricting to Thoroughbreds in the original GWA (*N* = 85), we found no association of chromosome 6 with anhidrosis. Collectively, these data do not support a role of the *KCNE4* variant or the GWA SNV in equine anhidrosis.

1

Anhidrosis in horses can be detrimental to their performance and wellbeing. A genome‐wide association (GWA) utilizing data from 200 horses (Thoroughbreds and stock horses (Quarter Horse, Appaloosa, Paint Horse)) genotyped on the Affymetrix 670 k Axiom Equine Array and using owner‐reported phenotypes, suggested that a region of chromosome 6 (NC_009149.3) was associated with the condition (Patterson Rosa et al. 2021). The authors then evaluated whole‐genome sequence (WGS) data from one control and one affected stock type horse and reported a missense variant ~148.5 kb from one of the single nucleotide variants (SNV) that was significantly associated with phenotype in the GWA. The authors proposed that variant, NC_009149.3:g.11813731A>G, found in the gene *KCNE4* (Potassium Voltage‐Gated Channel Subfamily E Regulatory Subunit 4), as a risk factor for the development of anhidrosis. This conclusion was highlighted by several equine‐related media outlets (e.g., Kentucky Equine Research 2022; Mallicote 2023; Moore 2021). Based on those findings, a genotyping company began offering a commercial test marketed to predict a horse's risk in developing anhidrosis. That test is assumed to be based on the genotype of the *KCNE4* SNV (NC_009149.3:g.11813731A>G) as the company's website states, the “Gene or Region: KCNE4,” and lists the alleles as A and G (https://etalondx.com/horse‐genetics/horse‐health/chronic‐idiopathic‐anhidrosis‐risk/; accessed 22 Aug 2025). The genotypes at NC_009149.3:g.11813731A>G were not studied in either the 200 horses utilized in the GWA or in an additional 131 horses with owner‐reported phenotypes described in the original publication (Patterson Rosa et al. 2021). The basis of the authors' conclusion that the *KCNE4* variant is a potential risk factor for anhidrosis was therefore based only upon the reported presence of the variant in the whole‐genome sequence data generated from two horses. The authors did acknowledge that further investigation of the variant in a larger sample of horses was required to validate its hypothesized role in anhidrosis.

van der Graaf et al. (2025) reported that, in a panel of 50 horses of various breeds, all 3 genotypes (A/A = 5, A/G = 27, G/G = 18) of NC_009149.3:g.11813731A>G were represented, with G as the major allele. That study also reported the G allele as the major allele in a query of public data of 820 horses. The high frequency of the allele is not compatible with its putative role conferring risk for an infrequent disorder. Upon publication, the result of van der Graaf et al. (2025) was questioned by the authors of Patterson Rosa et al. (2021), positing that the risk factor of *KCNE4* was the reference allele (A), not the alternate allele (G) of NC_009149.3:g.11813731A>G. We had assumed the G allele was the risk allele given HGVS nomenclature (e.g., reference allele>alternative allele), following which, NC_009149.3:g.11813731A>G defines the alternative allele as G. Our assumption was also driven by the protein model (figure 4 of Patterson Rosa et al. 2021), in which transcript XM_023642424.1 is referenced, describing the first panel of the figure as showing “the reference (CIA unaffected) sequence.” In transcript XM_023642424.1, the A allele is the reference allele.

Each of the 50 horses studied in van der Graaf et al. (2025) produced sweat using the standard sweat test, where different concentrations of terbutaline are administered intradermally and a sweat response assessed (Costa 2017). Further, the total sweat score (i.e., the sum of scores for the presence of sweat at each injection site) was not associated with the horses' genotype at the putative risk locus, regardless of which allele was considered the risk factor. While the results of the sweat test could have been further quantified by collecting the sweat on absorbent pads and weighing them, the semi‐quantitative assessment used in van der Graaf et al. (2025) produced clear results. The method of phenotyping by intradermal terbutaline injection is a higher standard than the owner‐reported phenotypes from survey data used in Patterson Rosa et al. (2021), which could easily result in incorrect classification of phenotypes.

An acknowledged weakness of the van der Graaf et al. (2025) publication was the lack of anhidrotic horses. Since publication, we received DNA samples on 24 additional horses evaluated by veterinarians for anhidrosis. The phenotype of 20 of these horses, all Thoroughbreds housed and managed in a similar environment in Florida, was classified using the quantitative intradermal terbutaline sweat test (QITST) following the method of Mackay (2008) utilizing five dilutions of terbutaline (0.1 mg/L, 1 mg/L, 10 mg/L, 100 mg/L, 1000 mg/L). A QITST score of 0 to 5 was assigned to each horse. This score indicated how many of the five injection sites had sweat weights below the 95% confidence interval limit, as established by Mackay (2008) in a Thoroughbred reference sample. Based upon their QITST scores, horses were then classified as free from anhidrosis (score of ≤ 1; *N* = 11), having partial anhidrosis (score of 2 or 3, *N* = 7), or having complete anhidrosis (score ≥ 4; *N* = 2). The other 4 samples (Quarter Horse (2), Clydesdale, and Warmblood) were clinically diagnosed anhidrosis cases (Table S1).

Sanger sequencing to genotype the *KCNE4* putative risk SNV was conducted on all 24 horses (Methods S1). Like the prior study, in the 24 new samples the G allele was the major allele (freq = 0.58) of locus NC_009149.3:g.11813731A>G. Considering only the 20 Thoroughbreds with QITST phenotypes, the overall frequency of the putative risk allele (A) was 0.425: 0.454 in the 11 control horses, 0.50 in the 7 horses with partial anhidrosis, and 0.0 in the 2 horses with complete anhidrosis (0.389 when combining horses with partial and complete anhidrosis). Fisher's Exact Tests determined that the A allele was not associated with the classification of the horses (free, partial anhidrosis, complete anhidrosis; *p* = 0.21). There was also no allelic association identified when considering the trait as binary (free vs. partial/complete anhidrosis; *p* = 0.76). As the commercial genotyping company states that horses heterozygous for the risk allele are at “moderate risk” and those homozygous at “high risk,” (https://etalondx.com/news‐media/genetic‐health‐risks‐chronic‐idiopathic‐anhidrosis/; accessed 22 Aug 2025), a dominant model (AA/AG vs. GG) was also considered but failed to identify an association of genotype with disease when considering the trait as binary (*p =* 0.16), or when splitting the anhidrotic horses into the classifications of partial or complete (*p* = 0.07). Finally, all three genotypes were present with the A allele the minor allele in the four clinical cases (genotypes: AA = 1, AG = 1, GG = 2).

Recent efforts of the animal genomics community are focused on classifying the potential pathogenicity of variants. Using the criteria of Boeykens et al. (2024), variant NC_009149.3:g.11813731A>G of *KCNE4* would be classified as benign with strong support due to criterion BS2 (i.e., observed in a healthy individual). Additionally, the variant is at high frequency when anhidrosis is relatively rare. Mayhew and Ferguson (1987) estimated anhidrosis occurred at a frequency of 6.1% in Thoroughbred horses in Florida, a hot, humid environment, where the disease is expected to be evident. A later estimate across breeds in Florida cited the incidence at 2% (Johnson et al. 2010). van der Graaf et al. (2025) found the frequency of the proposed risk allele (A) at NC_009149.3:g.11813731A>G as 0.44 in 286 Thoroughbreds and 0.32 in an across‐breed sample of 534 horses. The outcome of the sweat test of van der Graaf et al. (2025), the addition of data from QITST‐phenotyped Thoroughbred horses in Florida, the classification of the variant as benign, and the lack of NC_009149.3:g.11813731A>G genotypes for the owner‐reported anhidrotic horses used in the GWA (Patterson Rosa et al. 2021), indicate that the A allele of *KCNE4* is not a valid risk factor for anhidrosis.

Patterson Rosa et al. (2021) reported the putative risk allele of NC_009149.3:g.11813731A>G as a missense mutation. This annotation corresponds to the EquCab3 NCBI RefSeq Annotation (GCF_002863925.1‐RS_2024_08; Supplementary Table S2). While not provided in the original publication, based upon this annotation, a substitution of the reference A allele with a G allele would result in asparagine being replaced by serine at the 49th residue (XM_023642424.1:c.146A>G; XP_02348192.1:p.Asn49Ser). Referencing the same transcript, (XM_023642424.1), modelling in Patterson Rosa et al. (2021) was performed assuming an amino acid change in the 10th residue. If the annotation of Asn49Ser is correct, it is predicted by PhD‐SNP, PolyPhen‐1, PolyPhen‐2, and SIFT to be neutral, as determined in PredictSNP (Bendl et al. 2014). This computational prediction of impact also contributes to classification of the variant as benign per criterion BP4 of Boeykens et al. (2024). The annotation of the putative risk variant, however, does vary among genome annotations. Rather than being a missense variant as noted in the NCBI RefSeq annotation of both EquCab2 (GCF_000002305.2, 102, 2014‐11‐20) and EquCab3 (GCF_002863925.1‐RS_2024_08), the Ensembl annotation of EquCab3 (GCA_002863925.1; ver114.3) and the more recent Thoroughbred T2T assembly (GCF_041296265.1‐RS_2024_12) identify this variant as in the 5′ UTR of *KCNE4* (Table S2). The orthologous position is also in the 5′ UTR in both the human (GCF_000001405.40‐RS_2024_08; GRCh38.p14) and mouse (GCF_000001635.27‐RS_2024_02; GRCm39) assemblies. Based upon both the discrepancies in which amino acid is predicted to be altered by the variant and the annotation of the variant being in the UTR, the proposed impact of this variant on protein structure as reported in Patterson Rosa et al. (2021) needs to be reconsidered.

In addition to the putative causal variant, two additional SNVs in *KCNE4* were reported from WGS in Patterson Rosa et al. (2021) including a second missense variant of *KCNE4* and a synonymous variant within the gene. To better understand the genotypes at all three *KCNE4* variants, we downloaded the WGS data generated for and made available with the publication (BioProject PRJNA641747) and mapped the reads to the EquCab3 reference genome using BWA‐MEM (Li 2013). Upon inspection, we found both horses (the case and control) were homozygous for the reference (A) allele at the putative risk locus of *KCNE4* (NC_009149.3:g.11813731A>G; Table 1; Figure S1). Both horses were also homozygous for the EquCab3 reference allele (C) at the other missense locus (NC_009149.3:g.11814204C>A), and the control horse was heterozygous at the locus containing the synonymous variant (NC_009149.3:g.11813106G>A). A comparison of genome builds identified that the reference allele at each of the loci had changed between the prior reference (EquCab2) and EquCab3. EquCab3 was the reference with respect to which the *KCNE4* variants were reported in Patterson Rosa et al. (2021), although the notation of the reference allele used in the publication was not consistent across loci (Table S2). Still, the fact that both horses were homozygous for both missense variants of *KCNE4* brings into question the conclusion that NC_009149.3:g.11813731A>G is associated with the most significant GWA SNP or the condition itself.

**TABLE 1:** Genotypes (EquCab3) of the two horses whole‐genome sequenced by Patterson Rosa et al. (2021) for the GWA SNV and three reported *KCNE4* variants.

		Control	Case
		SAMN15364523	SAMN15364522
GWA Associated SNV	NC_009149.3:g.11665141C>T	CC	CT
*KCNE4* Synonymous	NC_009149.3:g.11813106G>A	GA	GG
**Proposed risk locus**	**NC_009149.3:g.11813731A>G**	**AA**	**AA**
*KCNE4* Missense	NC_009149.3:g.11814204C>A	CC	CC

*Note:* The proposed risk locus is shown in bold.

The question was then asked if the most significant hit from the GWA (“GWA SNV,” AX‐103822151, NC_009149.3:g.11665141C>T), rather than the *KCNE4* putative risk allele, might indicate risk for anhidrosis in the horses studied by van der Graaf et al. (2025) or in the new sample of Thoroughbreds. Using data from the original publication (Patterson Rosa et al. 2021), the positive predictive value of this marker was calculated to be only 63% in the 200 GWA horses. In the 50 horses studied by van der Graaf et al. (2025), the minor allele frequency of the GWA SNV was 0.45, with genotype frequencies of 0.30 (C/C), 0.50 (C/T), and 0.20 (T/T). Since all 50 horses produced sweat, there was no association of this allele or genotype at this locus and the ability to sweat in these horses. A Kruskall‐Wallace test also failed to identify an association of genotype at this locus with total sweat score (*p* = 0.71; Table 2). In the 20 new Thoroughbreds, there was no association of the allele at this locus with phenotype, or when considering a dominant genotypic model (Table S3). All 3 genotypes of the GWA SNV were also represented in the 4 clinical cases (CC = 1, CT = 2, TT = 1).

**TABLE 2:** Mean sweat score as calculated in van der Graaf et al. (2025) for each injection of terbutaline sulphate (mg/L) and mean total score by genotype at the GWA SNV (AX‐103822151).

Genotype (N)	Saline	mg/L terbutaline sulphate							
		0.001	0.01	0.1	1	10	100	1000	Total score (St Dev)
C/C (15)	0	0	0	0.670	0.400	0.933	1.333	1.800	6.67 (1.50)
C/T (25)	0	0	0	0.080	0.560	0.960	1.360	1.840	6.96 (1.58)
T/T (10)	0	0	0	0.000	0.400	1.000	1.300	1.800	6.70 (1.06)

*Note:* There was no difference in total sweat score by genotype (*p* = 0.71).

In the WGS data generated in Patterson Rosa et al. (2021), while both horses were homozygous A for the *KCNE4* putative risk variant, the case horse was heterozygous (C/T) for the GWA SNV, demonstrating incomplete linkage disequilibrium (LD) between GWA locus and the proposed risk locus. To further investigate LD between the GWA SNV and the *KCNE4* variants, WGS from a total of 897 horses was used, including data from Durward‐Akhurst et al. (2021), Bailey et al. (2024), Tozaki et al. (2021), the 50 horses studied in van der Graaf et al. (2025) and additional data from Donnelly et al. (2025); data from the 50 horses evaluated for anhidrosis were included in the latter reference. The horses included represented 26 breeds, several crosses, and some with unknown breed origin. If the GWA SNV was indeed tagging variation in *KCNE4* associated with risk for anhidrosis, LD between the two loci should be high.

To improve the accuracy of LD calculations, genotypes were first computationally phased utilizing WGS that included the loci of interest and flanking variation. Biallelic SNVs from the 897 horses, ranging from 50 kb upstream of the GWA SNV to 50 kb downstream of the most distal *KCNE4* SNV were extracted. After removing markers with a genotyping rate < 98% and minor allele frequency < 0.1, 588 SNVs spanning EquCab3 chromosome 6 (NC_009149.3) from 11 615 279 to 11 863 663 bp were included. Genotypes were phased using fastPhase (Scheet and Stephens 2006) with default settings. LD was calculated using haplotype and allele frequencies for all pairwise comparisons between the GWA locus, the putative risk locus, and the other missense locus in *KCNE4* as the correlation (*r* ^2^) described by Slatkin (2008). Across all horses, LD between the GWA locus and the putative risk locus was 0.19 (Table S4). The most significant GWAS SNV (AX‐103822151) of Patterson Rosa et al. (2021) is therefore not reliably tagging the proposed risk locus of *KCNE4* (NC_009149.3:g.11813731A>G). Linkage disequilibrium was similar between the GWA locus and that of the other missense variant in *KCNE4* (NC_009149.3:g.11814204C>A) as LD between the two *KCNE4* missense loci, separated by only 473 bp, was high (*r* ^2^ = 0.91).

Although the commercial test for anhidrosis is being marketed across breeds, Patterson Rosa et al. (2021) focused their study on stock horses and Thoroughbreds. Linkage disequilibrium between the GWA locus and the proposed risk locus, while negligible in the full data set and when considering only stock horses (*r* ^2^ = 0.06, *N* = 97), was moderate in Thoroughbreds alone (*r* ^2^ = 0.58; *N* = 369). It is well documented that LD in the Thoroughbred decays more slowly than in most other breeds (McCue et al. 2012). However, given the LD observed between loci in the Thoroughbreds, it was hypothesized that the GWA SNV may be tagging the candidate risk locus in that breed. A GWA was then performed using the Thoroughbred (designated as TA) genotype and phenotype data from the Patterson Rosa publication (38 cases, 47 controls). The annotation of the markers on the array was updated to the EquCab3 reference genome and the analysis conducted in GCTA (Yang et al. 2011) utilizing the fastGWA‐mlm‐binary model, which includes a sparse genomic relationship matrix. Five loci reached genome‐wide significance as determined by Bonferroni correction including two on chromosome 7 (Supplementary Figure S2), like the result reported in Patterson Rosa et al. (2021) utilizing their full dataset. Unlike their analysis across breeds, there were no significant associations on chromosome 6. The chromosome 6 SNV with the lowest *P*‐value in the analysis (AX‐104209117; *p* = 3.34E‐06) was over 61 Mb from *KCNE4*. Therefore, the association of chromosome 6 with owner‐reported anhidrosis was not replicated when considering only Thoroughbreds from that study, supporting the conclusion of the genotype analysis from the 20 Thoroughbreds phenotyped using the intradermal terbutaline sweat test.

In summary, like the results of van der Graaf et al. (2025), these data from Thoroughbred horses in a high‐risk environment fail to support the implication of *KCNE4* SNV NC_009149.3:g.11813731A>G as a risk factor for equine anhidrosis. The data also refute the association of the GWA SNV with disease. A GWA is expected to tag causative/risk loci. However, there was negligible LD between the most significant locus identified by GWA in Patterson Rosa et al. (2021) and the putative *KCNE4* risk locus. In Thoroughbreds, where moderate LD was present, we were unable to replicate a significant association of the GWA SNV with owner‐reported anhidrosis phenotypes. These additional analyses further question the original implication of the *KCNE4* putative risk locus given the identical genotypes of both the case and control horses sequenced, the lack of clarity of which allele was posited to be the risk factor, and its functional consequence. In total, the data do not support either the proposed missense variant of *KCNE4* or the GWA SNV as indicative of risk for the development of equine anhidrosis.

## Conflicts of Interest

A portion of the revenue generated by UC Davis Veterinary Genetics Lab (UCD‐VGL) for tests developed in the Finno Laboratory (MYHM and EJSCA) is returned to the Finno lab for research purposes for 5 years subsequent to their discovery. There is currently no genetic test for anhidrosis offered by the UCD‐VGL.

## Supporting information


**Figure S1:** Screenshot of Integrative Genomics Viewer (IGV) at the putative risk locus (NC_009149.3:11813731). The two samples shown are the whole‐genome sequence of the control (SAMN1536423, top) and affected (SAMN15364522, bottom) horses sequenced in Patterson Rosa et al. (2021). The A allele is present in all reads (39 in control, 52 in case) at the locus.

**Figure S2:** Manhattan plot resulting from a genome‐wide association of owner reported phenotype (anhidrosis) of 85 Thoroughbreds and 238 758 markers (data from Patterson Rosa et al. (2021)). The red line indicates genome‐wide significance as determined by a Bonferroni correction. No significant associations were found on chromosome 6. λ = 0.96.

**Table S1:** Age, sex, breed, phenotype and genotype of horses included. The amount of sweat produced at each dilution (mg/L) is given for the 20 Thoroughbreds. GWA SNV: NC_009149.3:g.11665141C>T, Putative risk locus: NC_009149.3:g.11813731A>G. G = gelding, M = mare.

**Table S2:** Information provided on four variants of focus in Patterson Rosa et al. (2021) including the GWA SNV and three variants in KCNE4. Given are genomic coordinates and annotation for several recent assemblies/annotations of the horse genome. The reference variant varies by annotation as does the consequence of the KCNE4 variants.

**Table S3:** Fisher's Exact Tests of phenotype and genotype based upon QITST testing of 20 Thoroughbreds for the GWA locus (NC_009149.3:g.11665141C>T). Allelic tests (C vs. T) and a dominance model (CC vs. CT and TT) were performed with anhidrosis being classified either into three categories (free, partial anhidrosis, complete anhidrosis) or as a binary trait (free vs. partial/complete anhidrosis).

**Table S4:** Frequency of each haplotype and linkage disequilibrium (r2) calculated from phased haplotypes considering: (A) the GWA SNP locus (AX‐103822151) and the putative risk locus (NC_009149.3:g.11813731A>G) for all horses, only Thoroughbreds, and only stock horses. The same information is given in (B), which considers LD between the GWA locus and the other missense locus of KCNE4 (NC_009149.3:g.11814204C>A), and (C), which evalutes the haplotype frequencies and linkage disequilibrium between the two putative missense loci in KCNE4.

**Data S1:** Supplementary Methods: Additional evidence fails to associate variation in KCNE4 with equine anhidrosis.

## Data Availability

The whole‐genome sequence of the 2 horses from Patterson Rosa et al. (2021) are available at the National Center for Biotechnology Information Sequence Read Archive (NCBI SRA) accessions PRJNA641747. Whole‐genome sequence of the 50 study horses of van der Graaf et al. (2025) and the additional horses from Donnelly et al. (2025) are available under accessions PRJNA841639, PRJNA553581 and PRJNA601992. The data utilized to calculate linkage disequilibrium are found at: Bioproject PRJNA993255 and accessions SRR19364580, SRR19364583, SRR19364585, SRR19364586, SRR19364587, SRR19364589, SRR19364593, SRR19364602, SRR19364605, SRR19364613, SRR19364619, SRR19364621, SRR19364627, SRR19364628, SRR19364629, SRR19364632, SRR19364641, SRR19364644, SRR19364645, SRR19364654, SRR19364658 (Bailey et al. 2024), and Bioproject PRJEB47918 (Durward‐Akhurst et al. 2021). Data from Tozaki et al. (2021) can be accessed through the Open Science Framework (https://doi.org/10.17605/OSF.IO/PVNCY).
