# Supplementary figures and images for: Additional Evidence Fails to Associate Variation in KCNE4 With Equine Anhidrosis
Source: Anim Genet. 2026 Apr 26;57:e70109. doi: 10.1002/age.70109 (PMC13110899; doi:10.1002/age.70109)

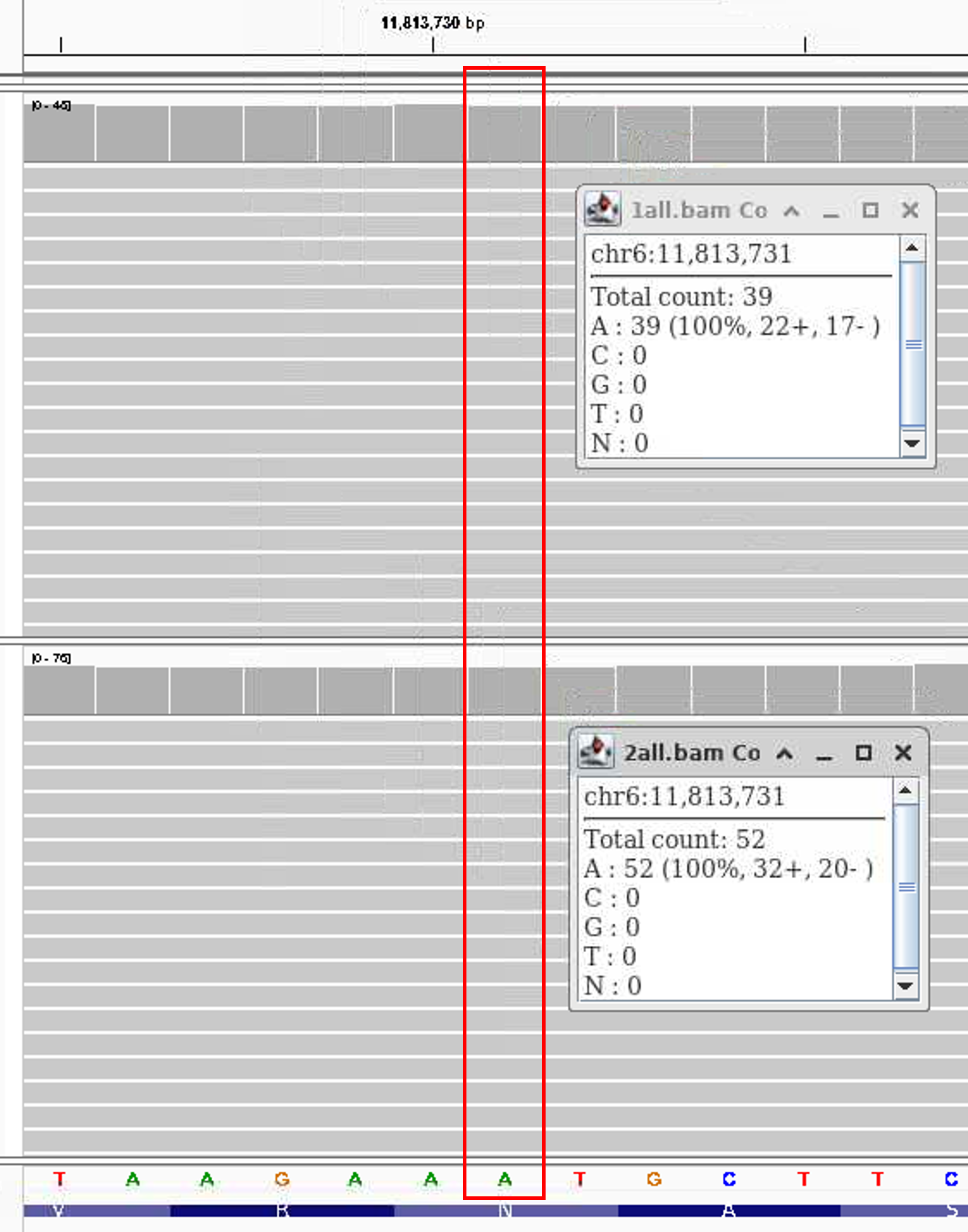

Supplement: Supplementary file 1 — Figure S1: Screenshot of Integrative Genomics Viewer (IGV) at the putative risk locus (NC_009149.3:11813731). The two samples shown are the whole‐genome sequence of the control (SAMN1536423, top) and affected (SAMN15364522, bottom) horses sequenced in Patterson Rosa et al. (2021). The A allele is present in all reads (39 in control, 52 in case) at the locus. [file AGE-57-0-s004.png]

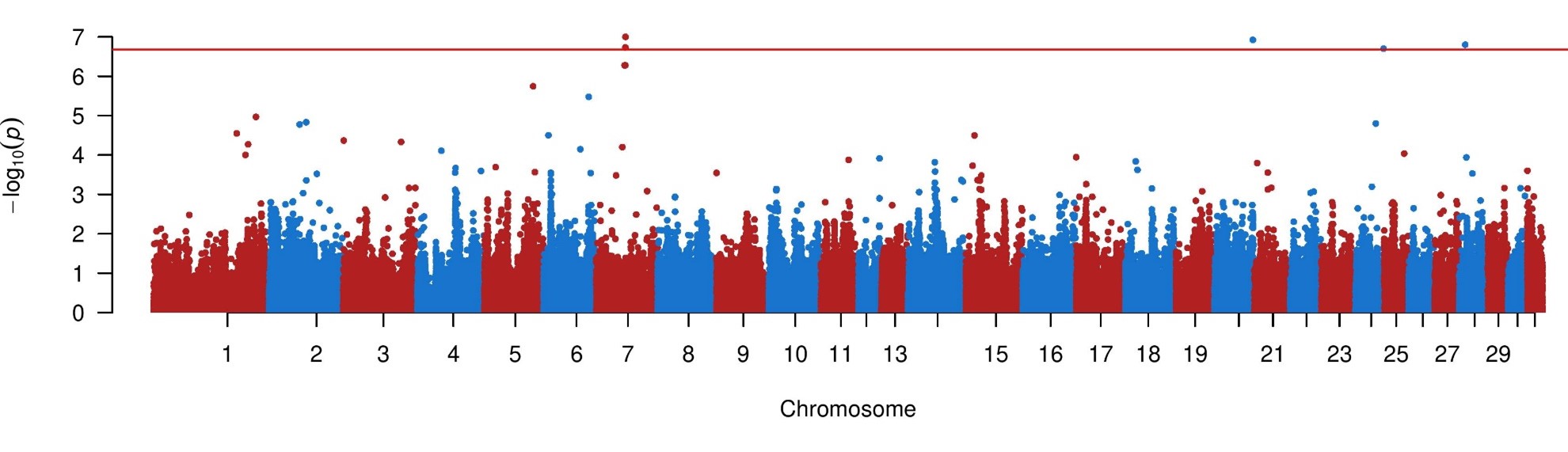

Supplement: Supplementary file 2 — Figure S2: Manhattan plot resulting from a genome‐wide association of owner reported phenotype (anhidrosis) of 85 Thoroughbreds and 238 758 markers (data from Patterson Rosa et al. (2021)). The red line indicates genome‐wide significance as determined by a Bonferroni correction. No significant associations were found on chromosome 6. λ = 0.96. [file AGE-57-0-s002.jpg]
