# Supplementary material for: Additional Evidence Fails to Associate Variation in KCNE4 With Equine Anhidrosis
Source: Anim Genet. 2026 Apr 26;57:e70109. doi: 10.1002/age.70109 (PMC13110899; doi:10.1002/age.70109)
Supplement: Supplementary file 7 — Data S1: Supplementary Methods: Additional evidence fails to associate variation in KCNE4 with equine anhidrosis. [file AGE-57-0-s007.pdf]

Supplementary Methods: Additional evidence fails to associate variation in *KCNE4* with equine anhidrosis

Genotyping of variants of interest by Sanger Sequencing

DNA was isolated from hair samples using the Quick-DNA Miniprep or Microprep Plus Kit (Zymo Research, Irvine, CA, USA) using the manufacturer's protocol. DNA was quantified using an Epoch 2 Microplate Reader (BioTek, Winooski, VT, USA).

Primers to amplify the the putative risk locus (NC_009149.3:g.11813731A>G) and the GWA SNV (NC_009149.3:g.11665141C>T) were designed based upon the EquCab3 reference genome in Primer3 (Untergasser *et al.*, 2012).

PCR reactions included 4µl of template DNA (5ng/µl), 2.4µl of GC-rich solution (5X), 0.25 µl MgCl, 1.2 µl 10X buffer with MgCl, and 0.5 µl of dNTP (10mM each), 0.5 units of FastStart Taq polymerase (Roche Diagnostics), 1.25 µl of each primer (20 µM each), and MilliQ water to volume.

The thermalcyler profile consisted of 94 °C for 4 min, 32 cycles of 94 °C for 30 sec, 58 °C for 30 sec, 72 °C for 45 sec, a final extension at 72 °C for 10 min, and hold at 10 °C. After checking PCR products on a 1.2% agarose gel, 3 µL PCR products were added to 0.75 µL ExoSAP-IT (Applied Biosystems, Foster City, CA, USA) and 13.25 µL MilliQ water. The samples were then subjected to the following thermalcycler conditions: 37 °C for 30 min, 80 °C for 15 min, and a hold at 15 °C. 2 µL of 20 µM primer was added before sending to Eurofins Genomics (Louisville, KY, USA) for Sanger sequencing on an ABI 3730xl. Sequencing results were analyzed using Sequencher 5.4.6 (Gene Codes Corporation, Ann Arbor, MI, USA).

Locus (NC_009149.3:g)	Primer 1 (5'-3')	Primer 2 (5'-3')	Fragment Size (bp)
11813731A>G	CATGCCCACTTTTATCAAATGCA	TCGTCTTTGTACAGCAGCAG	510
11665141C>T	TTTCTCCTTGAGCTGCAGGA	ACTAACACAGCGAAGGGGAA	503
